# Morphometric characteristics of the prefemoral fat pad of the knee on MRI: a study in a healthy population

**DOI:** 10.1186/s12880-026-02663-y

**Published:** 2026-08-12

**Authors:** Christoph Hellmund, Pierre Hepp, Hanno Steinke, Leon Weigelt, Timm Denecke, Jeanette Henkelmann

**Affiliations:** 1 Department of Orthopedics, Trauma and Plastic Surgery, University of Leipzig, Liebigstraße 20, Leipzig, 04103 Germany; 2 Institute of Anatomy, University of Leipzig, Leipzig, Germany; 3 Department of Diagnostic and Interventional Radiology, University of Leipzig, Leipzig, Germany

**Keywords:** Prefemoral fat pad, Infrapatellar fat pad, Volumetric segmentation, Insall-Salvati, Patellofemoral joint

## Abstract

**Background:**

The prefemoral fat pad (PFP) is located anterior to the distal femur and adjacent to the patellofemoral joint. While the infrapatellar fat pad (IFP) has been extensively studied, morphometric data on the PFP are scarce. The purpose of this study was to characterize the morphology of the PFP on MRI and to evaluate its relationship to adjacent osseous and soft tissue structures.

**Materials and methods:**

Twenty healthy volunteers were prospectively enrolled, and MRI examinations of 38 knees from 19 participants were analysed. Volumes of the PFP, IFP, and suprapatellar fat pad (SFP) were measured using manual segmentation. Additional morphometric parameters included body height, weight, BMI, age, numerous femoral and tibial osseous dimensions, the Insall-Salvati ratio, and the suprapatellar axial PFP area (SAPA). Correlation and regression analyses were performed to identify predictors and surrogate measures of PFP volume.

**Results:**

Mean PFP and IFP volume were comparable. PFP volume showed significant correlations with selected osseous parameters of the distal femur and proximal tibia. IFP volume and SAPA demonstrated a strong correlation with PFP volume and emerged as surrogate parameters. No significant relationship was found between PFP volume and patellar height as assessed by the Insall-Salvati ratio.

**Conclusions:**

In healthy adults, the PFP is similar in size to the IFP and shows consistent morphometric relationships with adjacent structures. SAPA may represent a simplified, time-efficient candidate surrogate measure that showed a strong association with total PFP volume in this healthy cohort; further validation in larger and symptomatic populations is required before it can substitute for complete volumetric segmentation in clinical practice. These findings provide baseline morphometric data for future studies investigating pathological alterations of the PFP.

## Background

**Fig. 1:**
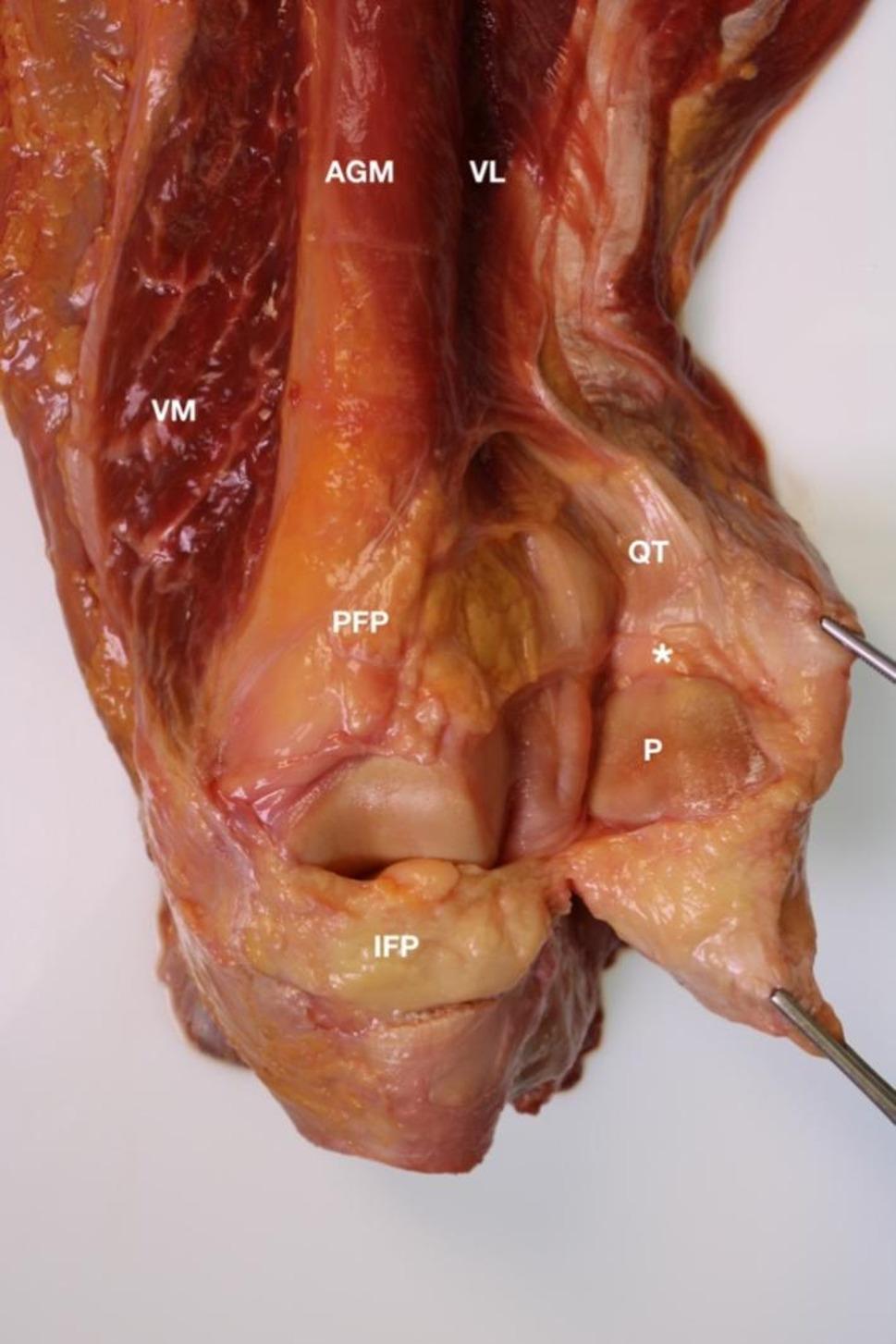
Anatomical dissection of the anterior knee fat pads. Coronal view of a cadaveric knee demonstrating the prefemoral fat pad (PFP) suprapatellar fat pad (*), and infrapatellar fat pad (IFP). The PFP is positioned anterior to the femoral condyle, posterior to the patella (P) and superior to the trochlear groove. It is closely related to the quadriceps tendon (QT), the lateral and medial vastus muscles (VL and VM) and the articularis genus muscle (AGM; reproduced from [1] with permission)

The prefemoral fat pad (PFP) is an underexplored intra-articular structure located posterior to the suprapatellar fat pad (SFP) and anterior to the femoral condyle [1] (Fig. 1). It is thought to act as a deformable adipose structure during knee motion, facilitating patellofemoral articulation and accommodating mechanical stress [2–5].

Emerging evidence suggests that the PFP may be involved in degenerative and inflammatory knee conditions, particularly osteoarthritis (OA). MRI-based studies have reported morphological alterations of the PFP, including edema and hypertrophy, which have been associated with pain and functional impairment in patients with OA [6, 7]. However, PFP edema is not specific to degenerative disease and has also been described in a variety of non-degenerative conditions, such as proliferative synovitis, HIV-related arthropathy, and even in asymptomatic athletes [7–11]. These observations suggest that signal alterations of the PFP on MRI alone are insufficient to reliably distinguish physiological from pathological states.

Similar to the infrapatellar fat pad (IFP), the PFP consists of highly innervated adipose tissue and may contribute to the intra-articular inflammatory environment of the knee [7, 12]. Adipose-derived cytokines and inflammatory mediators released from intra-articular fat pads have been increasingly studied in the context of degenerative joint disease, highlighting their potential role as modulators of local inflammation rather than passive space fillers [6]. Nevertheless, the specific biological contribution of the PFP remains incompletely understood, and imaging-based characterization is required to establish a structural framework for future functional and biochemical investigations.

Despite growing interest in the PFP, its basic morphological characteristics have not been systematically described. In contrast to the IFP, for which volumetric and morphometric reference data are available, information on PFP volume and its relationship to surrounding osseous and soft tissue structures is lacking, particularly in healthy individuals. The absence of normative morphometric data limits the interpretation of PFP alterations observed in pathological conditions and underscores the need for a detailed radiological characterization of the PFP in a healthy population. The purpose of this study was therefore to characterize the morphology of the PFP on MRI and to evaluate its relationship to adjacent structures.

## Materials and methods

### Study design and participants

This cross-sectional observational study investigated the anatomical and morphometric characteristics of the PFP using MRI. Twenty healthy volunteers (10 females, 10 males; mean age 23.8 ± 3.5 years) were prospectively enrolled. Anthropometric data including body weight, height, and body mass index (BMI) were recorded for all participants.

Inclusion criteria were the absence of current or previous knee pain, no history of knee trauma or surgery, and no known musculoskeletal or systemic conditions affecting the knee joint. Exclusion criteria included contraindications to MRI (e.g., metallic implants, cardiac pacemakers or event recorders, or severe claustrophobia).

Both knees of each participant were included, and the within-subject dependency of bilateral measurements was addressed using mixed-effects models with participant-specific random intercepts. The study protocol was approved by the Ethics Committee of the University of Leipzig (185/25-ek) and conducted in accordance with the Declaration of Helsinki (1964 and subsequent amendments). Written informed consent was obtained from all participants prior to the imaging procedure.

### MRI acquisition

MRI of both knees was performed using a 1.5-T scanner (Magnetom Aera, Siemens Healthineers, Erlangen, Germany) with a dedicated knee coil. All examinations were acquired with the knee positioned in approximately 15° of flexion using a standardized wedge to ensure reproducible patellofemoral alignment and optimal visualization of the prefemoral fat pad.

The imaging protocol included a proton density–weighted fat-suppressed coronal sequence (repetition time [TR] 3500 ms, echo time [TE] 36 ms, slice thickness 3.0 mm), which was used for anatomical orientation and assessment of surrounding joint structures. For morphometric analysis and volumetric segmentation, a high-resolution T1-weighted sagittal three-dimensional SPACE sequence was acquired (isotropic voxel size 0.63 mm, TR 600 ms, TE 14 ms), allowing multiplanar reconstructions of the patellofemoral joint.

In one participant, both knee examinations were excluded due to severe motion-related artifacts, resulting in a total of 38 knee datasets available for analysis.

### Image analysis

All MRI datasets were analyzed using Philips Advanced Visualization Workspace (Philips, Amsterdam, Netherlands). The three intraarticular extrasynovial fat pads of the knee (SFP, PFP and IFP) were identified on each scan. Volumetric measurements of all fat pads were performed manually on isotropic 3D T1-weighted sequences. All measurements were performed independently by two readers, blinded to participant characteristics: a junior physician with research experience in knee anatomy trained for MRI analysis, and a musculoskeletal radiologist with 11 years of experience. Inter- and intraobserver reliability was assessed using intraclass correlation coefficients (ICC), and discrepancies were resolved by consensus. Morphometric parameters recorded included: patellar length and patellar tendon length, Insall-Salvati ratio (ISR), femoral intercondylar distance, width of the tibial plateau, width of the femoral diaphysis and maximum height and maximum anterior-posterior depth of the PFP. Additionally, the directly suprapatellar axial PFP area (SAPA) was measured as a practical surrogate for total PFP volume, since the full PFP often extends cranially beyond the field of view of standard knee MRI sequences, making complete volumetric assessment challenging. SAPA was measured on axial images reconstructed from the isotropic 3D T1-weighted SPACE sequence at the level of the suprapatellar recess, defined as the axial plane immediately cranial to the point where the trochlear cartilage was no longer visible. To minimize partial-volume effects, the outline was drawn on the single axial slice showing the maximal cross-sectional PFP area rather than averaged across adjacent slices, with boundaries placed at the outer margin of the visible fat signal. The articularis genus muscle and other intrafat muscular strands were consistently excluded from the outlined area in all measurements (Figs. 1 and 3). Vessels within the PFP were identified on high-resolution T1-weighted images as tubular, linear structures of low signal intensity coursing through the fat pad, distinguishable from adjacent muscle strands by their smaller caliber, curvilinear course, and lack of continuity with adjacent muscle bellies (Fig. 3).

**Fig. 2:**
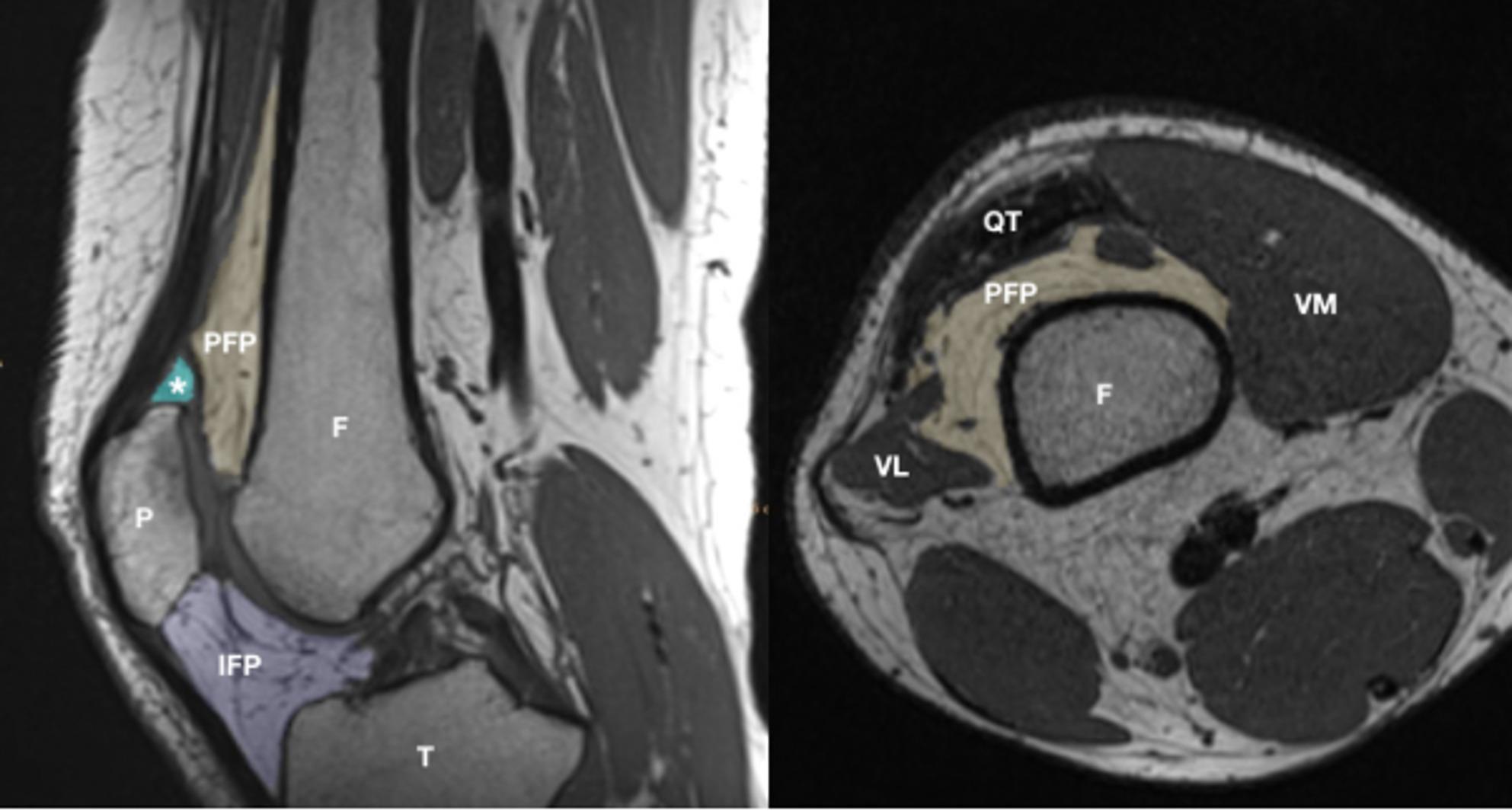
T1-weighted MRI scan in the sagittal and axial plane of the prefemoral fat pad (PFP, yellow). The infrapatellar fat pad (IFP, purple) and the suprapatellar fat pad (SFP *, blue). PFP Boundaries are defined as the femur (F) dorsally, the quadriceps tendon (QT) and suprapatellar recess ventrally, the trochlear cartilage distally, the lateral border of the medial vastus muscle (VM) medially and the medial border of the lateral vastus muscle (VL) laterally

### Statistical analysis

Statistical analyses were conducted using IBM SPSS Statistics 29 (IBM, New York, USA). Continuous variables are presented as mean ± standard deviation. Normality of distribution was assessed using the Shapiro–Wilk test. As PFP volume was not normally distributed, Spearman’s rank correlation was used to examine associations with other morphometric parameters.

For regression analyses, PFP volume and SAPA were log-transformed to improve normality, and all variables were standardized using Z-scores to allow for direct comparison across different measurement scales. To account for the inclusion of both knees per participant, linear mixed-effects models (LMM) were computed with participant-specific random intercepts, and sex, side (left/right), and relevant morphometric variables as fixed effects. Multicollinearity was assessed using variance inflation factors (VIF), with a threshold of > 5 indicating potential collinearity.

A second, reduced LMM was calculated including only sex, side, and standardized IFP volume, SAPA, and ISR as fixed effects. This model focused on the key variables identified in the full LMM to reduce the risk of overfitting and highlight the most relevant predictors.

To further assess within-participant associations, right-left difference scores for both outcomes and predictors were analyzed using difference-score regression (Δ-OLS).

Additionally, PFP volume differences according to ISR were evaluated using the Mann–Whitney U test as an exploratory analysis. Model assumptions were verified through visual inspection of residuals. A two-sided p-value < 0.05 was considered statistically significant.

**Fig. 3:**
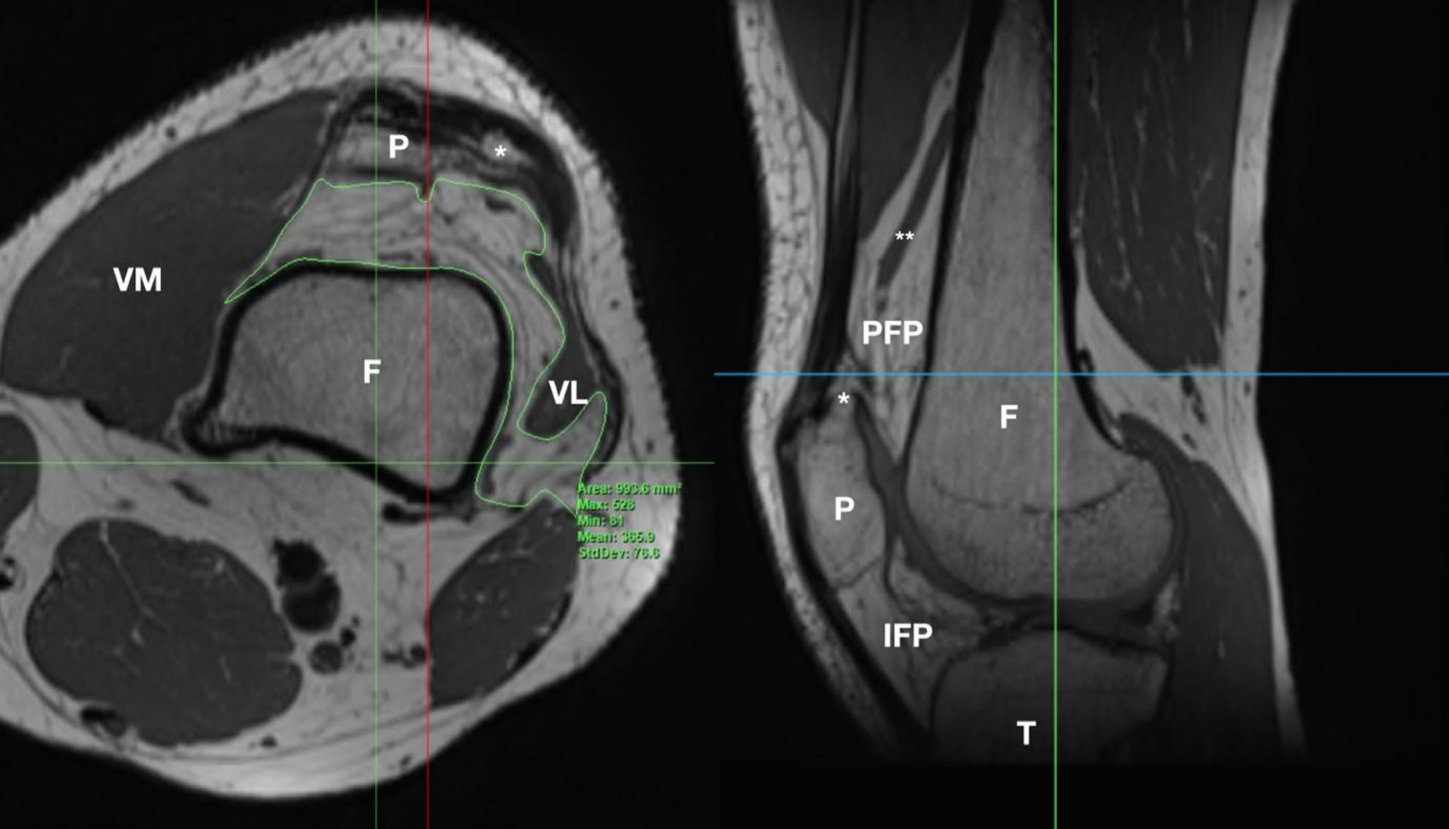
Suprapatellar axial PFP area (SAPA) measurement. Left: Axial MRI at the level of the suprapatellar recess demonstrating the outlined PFP area (SAPA, green). The PFP lies adjacent to the medial and lateral vastus muscles (VM and VL), the femur (F) and the patella (P). The SFP (*) is separated from the PFP via the suprapatellar recess. Right: median sagittal view of the knee. The blue line indicates the level of the corresponding axial view. The PFP lies posterior to the patella (P) and the SFP (*) and anterior to the femur (F). The articularis genus muscle (**) extends into the PFP and its strands were excluded from SAPA area measurement. The PFP does not communicate directly with the IFP or the tibia (T)

## Results

The mean body weight of the cohort was 67.8 ± 11.1 kg and the mean height 172.1 ± 8.6 cm, the mean BMI was 22.74 ± 2.16 kg/m^2^.

All three intra-articular, extrasynovial fat pads (PFP, IFP and SFP) were identifiable in all 38 MRI datasets. The anatomical boundaries of the PFP were as follows: distally by the proximal trochlear cartilage, medially by the lateral border of the vastus medialis, laterally by the lateral intermuscular septum or, if this was not clearly identifiable (~ 10% of cases), by an imaginary line connecting the dorsolateral femoral edge and the posterior border of the vastus lateralis, dorsally by the femur, and ventrally by the quadriceps tendon and superior recess. Proximally, the PFP tapered and became indistinct at approximately 10 cm above the trochlear cartilage (Fig. 2).

Intra-PFP vessels were visible in all scans. The AGM traversed the PFP in 92% of knees and inserted into the superior recess; in the remaining 8%, the muscle ended within the PFP. For suprapatellar axial PFP area (SAPA) measurements, strands of the AGM were excluded (Fig. 3).

**Fig. 4:**
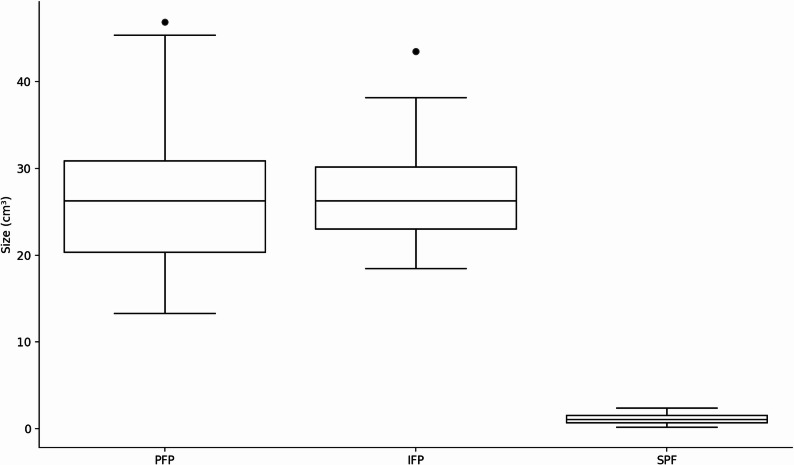
Boxplot illustrating the volume distribution of the infrapatellar fat pad (IFP), prefemoral fat pad (PFP), and suprapatellar fat pad (SFP) across 38 healthy knees. Median, interquartile range, and outliers are indicated

Volumetric analysis showed that the IFP was the largest fat pad (27.40 ± 5.96 cm³), followed closely by the PFP (26.67 ± 8.73 cm³). The SFP was smallest (1.12 ± 0.54 cm³) (Fig. 4). Median PFP volume was 25.5 cm³ (IQR 19.8–31.3 cm³). The mean ISR was 1.19 ± 0.20; 16 knees had ISR > 1.2, and 22 knees had ISR between 0.8 and 1.2. No knees had ISR < 0.8.

The Shapiro–Wilk test indicated non-normal distribution of PFP volumes (W = 0.93, *p* = 0.019). Spearman correlation analysis revealed strong positive associations between PFP volume and SAPA (ρ = 0.74, *p* < 0.001), maximum PFP height (ρ = 0.71, *p* < 0.001), SFP volume (ρ = 0.67, *p* < 0.001), weight (ρ = 0.64, *p* < 0.001), and IFP volume (ρ = 0.62, *p* < 0.001) (Table 1). Moderate correlations were observed with maximum anteroposterior PFP depth (ρ = 0.60, *p* < 0.001), BMI (ρ = 0.56, *p* < 0.001), tibial plateau width (ρ = 0.54, *p* < 0.001), and intercondylar distance (ρ = 0.53, *p* < 0.001). Body height, femoral diaphyseal width and age showed a weak correlation (ρ = 0.44, *p* = 0.006, ρ = 0.33, *p* = 0.04 and ρ = 0.3, *p* = 0.06, respectively), while PFP volume was not correlated with the ISR (ρ = 0.06, *p* = 0.73).

**Table 1:** Spearman correlation coefficients (ρ) between prefemoral fat pad (PFP) volume and investigated morphometric and anthropometric variables

Variable	Spearman ρ	*p*-value
SAPA	0.74	< 0.001
Maximum PFP height	0.71	< 0.001
SFP volume	0.67	< 0.001
Body weight	0.64	< 0.001
IFP volume	0.62	< 0.001
Maximum anteroposterior PFP depth	0.60	< 0.001
BMI	0.56	< 0.001
Tibial plateau width	0.54	< 0.001
Femoral intercondylar distance	0.53	< 0.001
Body height	0.44	0.006
Femoral diaphyseal width	0.33	0.04
Age	0.3	0.06
Insall-Salvati ratio (ISR)	0.06	0.73

Measurement reliability was high, with an ICC(1) of 0.91 (95% CI 0.78–0.96). The initial linear mixed-effects model (LMM) identified IFP volume, SAPA, patellar tendon length, and ISR as statistically significant predictors out of all variables. Due to collinearity between ISR and patellar tendon length (VIF > 10), a reduced LMM was fitted including sex, side, and standardized IFP volume, SAPA, and ISR. In this model, PFP volume was significantly associated with IFP volume (β = 0.13, SE = 0.04, *p* = 0.002) and SAPA (β = 0.01, SE = 0.04, *p* = 0.03), whereas sex, side, and ISR showed no significant effects. The random intercept variance was 0.43. Δ-OLS regression confirmed IFP volume as the predominant predictor of within-participant differences in PFP volume (β = 0.03, SE = 0.008, *p* = 0.006).

Exploratory comparison of PFP volume by patellar height indicated that knees with patella alta (ISR > 1.2, *n* = 16) had a median PFP volume of 27.4 cm³ (IQR 21.3–34.4), while knees with ISR 0.8–1.2 (*n* = 22) had a median volume of 21.9 cm³ (IQR 19.6–30.3); this difference was not statistically significant (*p* = 0.359, Mann–Whitney U test).

## Discussion

The primary finding of this study is that in healthy adults, the PFP is approximately as large as the IFP, with a mean volume of 26.7 cm³, but with greater inter-individual variability. This confirms that the PFP is a substantial intra-articular structure. Among all measured variables, IFP volume and SAPA were the strongest independent predictors of total PFP volume in multivariable modeling. Several other structural factors, including body weight, BMI, femoral and tibial morphology, patellar parameters, and SFP volume, showed significant positive associations with PFP volume in bivariate correlation analyses; however, their independent contribution was attenuated once IFP volume and SAPA were accounted for in the multivariable model.

Anatomically, the PFP lies anterior to the femur, superior to the trochlear groove, and adjacent to both the SFP and IFP (Figs. 1, 2 and 3) [1]. It contains key vascular structures, including the superior medial genicular and descending genicular arteries, which may be relevant in hypervascularized conditions and procedures such as transarterial periarticular embolization (TAPE) [13]. Its compliance and lobulated adipocyte composition likely allow it to act as a cushion and gliding structure during patellofemoral motion.

Previous studies in OA patients have shown reduced maximum anterior posterior depth and decreased deformation of the PFP during quadriceps contraction, suggestive of PFP fibrosis, as well as associations with decreased knee flexion and anterior knee pain [5, 7, 14]. Edema of the PFP has been observed in OA, proliferative synovitis, HIV infection, and even in asymptomatic athletes, indicating that edema alone is an unreliable marker of pathology [8–11]. Our findings in healthy adults suggest that PFP volume is primarily determined by local anatomical factors rather than patellar alignment, in contrast to the IFP, which appears more sensitive to patellar tendon length and height [15].

In this cohort, knees with patella alta (ISR > 1.2) had slightly larger median PFP volumes than those with normal ISR (0.8–1.2), but the difference was not statistically significant. This likely reflects the small, asymptomatic sample and absence of edema or pathology and larger cohorts are needed to validate our findings in more heterogeneous populations or symptomatic individuals. Prior studies suggest that symptomatic PFP edema may be associated with higher ISR, altered trochlear geometry, and increased lateral patellar tilt, but the relationship between PFP volume or edema and patellar stability remains inconclusive [16–18].

SAPA showed a strong association with total PFP volume, even after accounting for other morphometric variables in the multivariable model and therefore represents a promising simplified morphometric parameter. In this healthy cohort, SAPA could be regarded as a candidate surrogate measure for total PFP volume; however, its diagnostic performance, prediction error, longitudinal responsiveness, and clinical utility in symptomatic or pathological populations have not yet been established. Dedicated validation studies are required before SAPA can be recommended as a substitute for full volumetric segmentation in routine clinical practice.

Our findings are particularly interesting in the context of different functional roles of the fat pads of the knee. While the IFP is known to be a major mediator of proprioceptive and inflammatory responses, the SFP is accepted to serve as an important gliding structure [19, 20]. The primary PFP however has not been fully understood. Our analysis shows that the PFP is volumetrically comparable to the IFP.

Given its position between patella, femur, and suprapatellar bursa, and its capacity for deformation, we hypothesize that the PFP could act as a dynamic stabilizer within the anterior knee, analogous to proposed roles of other intra-articular fat pads. This hypothesis is further supported by the previous histological identification of proprioceptive structures within the PFP [1]. However, the present cross-sectional morphometric data in healthy adults cannot establish a functional or dynamic role for the PFP, and this concept remains speculative pending dedicated biomechanical and functional studies.

The relatively small cohort (19 participants, 38 knees) limits the generalizability of predictive estimates. The inclusion of both knees per participant introduces within-subject dependency; however, this was appropriately addressed using mixed-effects modeling and random participant intercept. While upper confidence limits for predictive estimates remain above 1, the present data still provide robust normative reference values for the PFP and establish methodological standards for future studies.

In summary, these results demonstrate that even with a moderate sample size, meaningful anatomical and morphometric insights can be derived in this healthy cohort. SAPA emerged as a promising candidate surrogate measure for PFP volume. Its potential application in the research and clinical evaluation of anterior knee pain, patellofemoral disorders, and osteoarthritis represents a hypothesis-generating future research direction requiring validation in larger and symptomatic populations, rather than a direct implication of the present findings. Future work should focus on validating SAPA in larger and symptomatic cohorts to confirm its predictive value for clinical outcomes. Furthermore, future studies should investigate PFP characteristics under dynamic and functional conditions.

## Conclusion

The PFP is a substantial intra-articular knee structure, comparable in size to the IFP, and its volume is primarily determined by local anatomical factors in this healthy cohort. SAPA demonstrated a strong association with total PFP volume and represents a promising, simplified candidate surrogate measure; however, further validation in larger and symptomatic populations is required before SAPA can be applied as a substitute for full volumetric segmentation in clinical assessment. These findings provide normative reference data and a methodological foundation for future research investigating PFP hypertrophy, edema, and their potential role in anterior knee pain and patellofemoral pathology.

## Data Availability

The data that support the findings of this study are available from the corresponding author upon reasonable request.

## Abbreviations

PFP: Prefemoral fat pad
IFP: Infrapatellar fat pad
SFP: Suprapatellar fat pad
SAPA: Suprapatellar axial PFP area
AGM: Articularis genus muscle
ISR: Insall-Salvati ratio
